# Mortality and morbidity in COVID-19 orthopedic trauma patients: is early surgery the keystone?

**DOI:** 10.11604/pamj.2021.38.163.27125

**Published:** 2021-02-12

**Authors:** Pietro Domenico Giorgi, Enrico Gallazzi, Paolo Capitani, Elena Biancardi, Federico Bove, Umberto Mezzadri, Dario Capitani, Giuseppe Rosario Schirò

**Affiliations:** 1 Department of Orthopedic Surgery and Traumatology, ASST GOM Niguarda, Milan, Italy

**Keywords:** COVID-19, fractures, mortality, early surgery, outcomes, ASA grade

## Abstract

In the pandemic disease caused by SARS-CoV-2 virus, trauma surgery continued the management of patients with fractures. The purpose of the study is to evaluate mortality and morbidity in orthopedic trauma patients surgically treated with a diagnosis of COVID-19 infection, comparing them to a control group of COVID-19 negative. We retrospectively identified patients admitted to our Emergency Room from March 8^th^ to May 4^th^ 2020 (time frame corresponding to the first wave of the pandemic peak, one of the most severe in the world at that time) with a diagnosis of fracture that were subsequently surgically treated. We applied a dedicated pathway for the management of COVID-19 trauma patients allowed to perform an early surgery and short hospitalization. For each patient included demographics, clinical, laboratory, radiological data and type of treatment for COVID-19 infection were collected. Sixty-five (65) patients were identified. Of those, 17 (6 women and 11 men, mean age 63.41 years old, mean ASA grade 2.35) were COVID-19 positive (study group), while the others were control group (mean age 56.58 years old, mean ASA grade 2.21). In the study group, the preoperative laboratory tests showed leukocytosis in six and lymphopenia in 15 cases. Fourteen patients had a high level of C-reactive protein. Fifteen patients had an abnormal level of D-dimer. The mortality recorded was 5.8% and 4.1% in the study and control group respectively. Perioperative adverse events were registered in 5 cases (29.4%) in the study group and in 8 (16.6%) in the control group (p>0.05). Dedicated COVID-19 trauma pathway with the aim of an early surgery could be key for a better result in terms of mortality and morbidity. Age and ASA grade could represent independent risk factors for perioperative complications.

## Introduction

The SARS-CoV-2 virus from the Hubei region of China has spread all over the world becoming a pandemic disease, according to the WHO. Its impact has been devastating and has plunged many health systems around the world [1]. The need for new dedicated COVID-wards and novel intensive care beds than those available required a reorganization of both the single hospitals and the regional network. In Italy, hospitals suspended the elective surgical activity and continued only with surgical emergencies [2]. Trauma surgery continued the management of patients with fractures by adapting to protocols of COVID-19's identification and prevention. Patients with severe fractures require surgical treatments and hospitalization that could become prolonged and therefore expose to hospital infections and complications. In a historical period in which the virus circulates among the people with a high rate of contagiousness, the risk of contracting the infection in these patients is high. It is also not uncommon to find patients that access the emergency room for various diseases, including traumas and fractures, in which COVID-19 infection is moreover found [3]. In literature, there is a lack of clinical studies that evaluate prognosis and complications of COVID-19 positive patients with fracture treated with orthopedic surgery [4]. The purpose of the study is to evaluate mortality and morbidity in orthopedic trauma patients surgically treated with a diagnosis of COVID-19 infection, comparing them to a control group of COVID-19 negative.

## Methods

**Study population:** we retrospectively identified all patients admitted to our Emergency Room (ER) from March 8^th^ to May 4^th^ 2020 with a diagnosis of fracture that were subsequently surgically treated by the orthopedic team. The time span selection refers to the duration of the phase I of the national COVID-19 outbreak response, during which the population was quarantined and movements were allowed only for urgent or essential reasons. Exclusion criteria were: age <18 years old, pathological fractures and polytrauma patients. In the study group, we inserted all patients positive for COVID-19 infection at admission or that become positive in less than 14 days after the admission (incubation period). All the other patients were identified as the control group.

**Figure 1:**
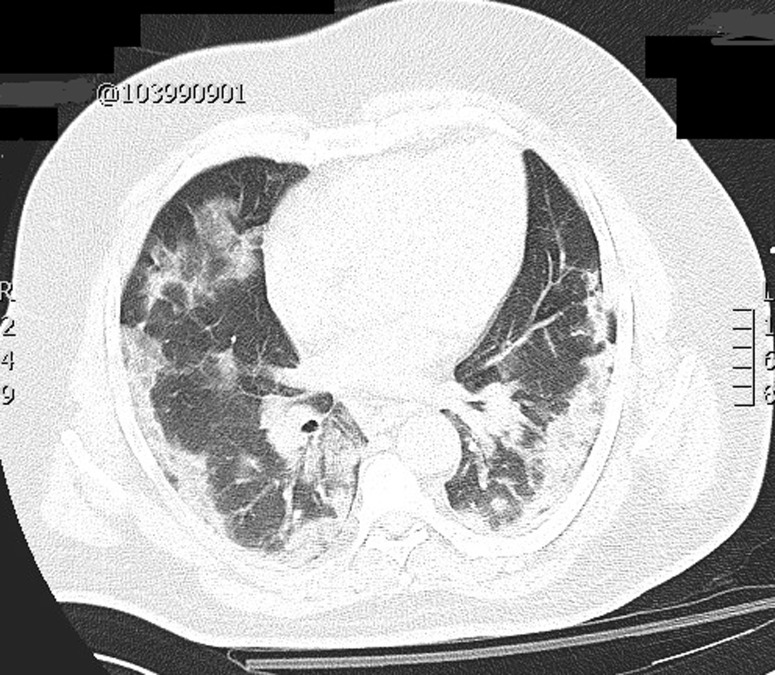
chest CT of a patient COVID positive with the typical interstitial pneumonia

**Management and diagnosis of COVID-19 infection:** to rationalize the use of resources and to reduce the risk of COVID-19 infection in hospital a management trauma flow-chart with different pathways for COVID-19 negative and positive patients was applied. Each patient with a trauma triage is tested for COVID-19 at admission, unless they have a recent (<7 days) negative test. For the purpose of this study, a patient was considered COVID-19 positive according to the molecular test that identifies the presence of the virus in the upper airways on the basis of quantitative reverse transcription of polymerase chain reaction (qRT-PCR). Moreover, diagnosis was confirmed with a High Resolution-computed tomography scan (HR-CT scan) of the thorax, used to identify the typical interstitial pneumonia (Figure 1). Molecular tests were repeated during the hospital stay in case of new respiratory symptoms, unexplained fever or any clinical finding suggestive for infection. Three different trauma pathways were set up in our emergency room: a ‘green’ clean pathway for known COVID-19 negative patients, a ‘red’, dirty for known positive and a ‘grey’ for unknown status waiting for the test results. These ‘grey’ patients, once the results of their test are available, follow either the ‘red’ or ‘green’ pathway for the diagnostic and therapeutic workup. Patients requiring urgent early surgery are treated as positive until the definitive test results are available [5].

**COVID-19 trauma pathway:** in COVID-19 trauma patients, surgery could not be postponed or delayed. The basic principle to follow during the pandemic is to limit as much as possible the hospital stay. In this context, the surgical timing plays a crucial role; our goal is to treat all patients in the “golden 72 hours”. To hit the target an orthopaedic trauma surgical team was made available for emergencies 24/7, using a dedicated operative room. To fit the system, all trauma emergencies have been classified according to the urgency of the condition and assigned to a color code. Each color is associated with a time, in hours, within which the surgery should be performed. The urgencies´ stratification, filling in a form with patient´s health data and hanging it on a blackboard at the entrance to the operating room: surgical procedures are scheduled according to the priority given by the color code.

**Data collection and endpoints:** for each patient included, demographics, clinical, laboratory (complete blood count (CBC) and differential at the time of surgery), radiological data and type of treatment for COVID-19 infection were collected. In particular, the American Society of Anesthesiologists (ASA) grade and time interval (ΔT) between admission and surgery were evaluated and used for complication risk stratification. Perioperative mortality was our primary endpoint, perioperative adverse event was our secondary endpoint. These endpoints were assessed comparing positive (study group) and negative (control group) COVID-19 patients surgically treated in the same time span.

**Statistical analysis:** descriptive statistics were used to summarized the data. Categorical data were analysed using Fisher´s exact test; continuous data were compared using Student´s t test (http://graphpad.com/). P-values of less than 0.05 were considered statistically significant.

**Compliance with ethical standards:** all procedures followed were in accordance with the ethical standards of the responsible committee on human experimentation (institutional and national) and with the Helsinki Declaration of 1975, as revised in 2008. Informed consent was obtained from all patients for being included in the study.

## Results

**Figure 2:**
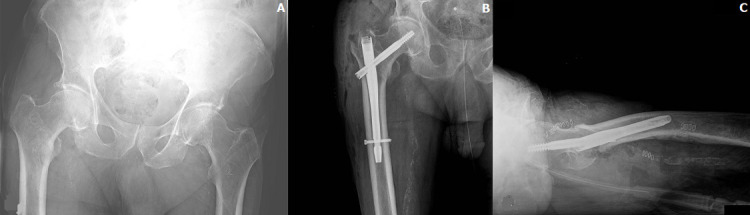
pre- (A) and post-operative (B, C) X-rays of a patient COVID positive with a fracture of the proximal femur

From the analysis of our institutional database, 65 patients were identified. Of those, 17 (6 women and 11 men) were considered COVID-19 positive at the time of surgery and formed the study group, while the others were used as a control group (Figure 2). The two groups were similar in terms of age, sex, ASA grade, fractures distribution and grade and time interval (ΔT) between admission and surgery (Table 1). Fracture type according to AO/OTA classification, surgical procedure, comorbidities and ASA grade for each patient in the study group are reported in Table 2.

**Table 1:** demographics, ASA grade, fracture types and time interval between admission and surgery between the two groups

	COVID Positive	COVID Negative	p
**Age**	63.41 ± 18.77	56.58 ± 25.27	ns
**Sex**	40.8% F, 59.2% M	35.3% F, 64.7% M	ns
**ASA grade**	2.35 ± 0.78	2.21 ± 0.81	ns
**Fracture type**	35.2% Vertebral Fracture; 29.4% Femoral Neck; 17.6% Distal Radius; 11.8% Humeral Fracture; 5.9% Proximal Tibia	28.6% Femoral Neck; 18.4% Vertebral Fracture; 12.2% Clavicular Fracture; 10.2% Distal Tibia/Malleoli; 8.2% Humeral Fracture; 8.2% Pelvis Fracture; 8.2% Femoral Shaft; 2% Calcaneus Fracture; 2% Metatarsal Fracture; 2% Distal Radius	
**Time interval admission to surgery (days)**	2.59 ± 2.91	2.54 ± 2.53	ns

ns: not significative

**Table 2:** age, type of fractures, classification and surgery, ASA grade and comorbidities for each patient in the study group

Patient	Age	Fracture Type	AO/OTA Classification	Type of Surgery	ASA Grade	Comorbidities
1	42	Distal Radius	23B2	ORIF	I	None
2	49	Distal Radius	23A2.2	ORIF	II	None
3	56	Humeral Shaft	12A1	Intramedullary Nailing	II	None
4	70	Perthrocantheric Fracture	31A2	Intramedullary Nailing	III	COPD, hypertension
5	80	Perthrocantheric Fracture	31A1	Intramedullary Nailing	II	None
6	81	Femoral Neck Fracture		Hemiarthroplasty	III	Diabetes, Hypertension, Alzheimer
7	88	Perthrocantheric Fracture	31A2	Intramedullary Nailing	III	COPD, hypertension, Diabetes, Atrial fibrillation
8	82	Humeral Shaft	12A2	Intramedullary Nailing	II	COPD
9	34	Distal Radius	23B2	ORIF	I	None
10	56	Perthrocantheric Fracture	31A2	Intramedullary Nailing	III	COPD
11	92	Proximal Tibia	41B3	ORIF	III	CLL, Hypertension, Chronic kidney disease
12	47	T5 Fracture	B2	T2-T7 Posterior Fixation	II	Hypertension, obesity
13	56	C4-5 Fracture	B2	ACCF C3-6	II	Hypertension, obesity
14	55	C5-6 Fracture	B3	ACCF C4-7	IV	Hypertension, Ischemic cardiopathy, Linfoma
15	35	C3-4 Fracture	B3	ACCF C2-5	II	Tuberculosis
16	77	C1-2 Fracture	A	C1-2 Fixation	III	Diabetes, MGUS, Rheumatoid arthritis
17	78	L1 Fracture	B2	T12-L2 Circumferential Fusion	II	Myeloma and breast cancer


ORIF: Open Reduction and Internal Fixation; ACCF: Anterior Cervical Corpectomy and Fusion; COPD: Chronic Obstructive Pulmonary Disease; CLL: Chronic Lymphocytic Leukemia; MGUS: Monoclonal Gammopathy of Undetermined Significance

Twelve patients were positive for molecular test for the coronavirus SARS-CoV-2 at the admission. Five patients had a negative admission test, but two of them had a thorax HR-CT scan compatible with SARS-CoV-2 and in fact developed a positive test during the hospital stay; three were found positive after surgery as a result of a longer incubation period. Concerning the clinical status related to the infection, four patients reported fever, cough, shortness of breath and diarrhea before hospitalization; ten patients had dyspnea and low saturation rate during the hospital stay that required O2 supplementation; three patients did not exhibit severe respiratory symptoms. Preoperative laboratory tests showed leukocytosis in six and lymphopenia in 15 cases. Blood coagulation assays showed normal activated partial thromboplastin time (APTT) in 14 patients and normal prothrombin time (PT) in all patients. However, 15 patients had an abnormal level of D-dimer. Fourteen patients had a high level of C-reactive protein, with four having high levels of procalcitonin. Therapy for COVID-19 infection with hydroxychloroquine and azithromycin was prescribed in six cases; in two of those, hydroxychloroquine was stopped due to liver toxicity. Three patients died with mortality of 5.8% and 4,1% in the study group (1 patient) and control group (2 patients) respectively (Table 3). The death in the study group was a direct consequence of COVID-19 infection with acute respiratory distress syndrome (ARDS). In the control group, one patient died for cardiac arrest while the other developed a respiratory insufficiency in a traumatic pulmonary edema. No correlation was found between mortality and the blood serum levels analyzed.

**Table 3:** clinical and biochemical data in the death patients

COVID-19	Sex, age	ASA grade	Diagnosis	Comorbidities	d-dimer	Leukocytes (10^9/L)	Lymphocytes	PT (seconds)	PTT (seconds)	PCR (mg/dl)	Procalcitonin (ng/ml)	Saturation, ventilation	Type of Surgery	ΔT Admission to Surgery (Days)
Yes	M, 92	III	Tibial plateau fracture	CLL, Hypertension, Chronic kidney disease	1.5	220	0.84	14	27.2	0.2	0.81	100% 6 L/min	ORIF	1
No	F, 71	IV	L1 Fracture	Depression/anxiety	0.7	20.1	0.69	16	28.7	0.3	0.77	100% 4 L/min	T12-L2 Posterior Fixation	0
No	F, 84	III	Pertrochanteric fracture	COPD, cardiopathy, Chronic kidney disease, Hypertension, ictus, DM	1.1	16.95	3.8	12	30.2	0.5	0.91	100% 4 L/min	ORIF	2

ORIF: Open Reduction and Internal Fixation; COPD: Chronic Obstructive Pulmonary Disease; CLL: Chronic Lymphocytic Leukemia; DM: Diabetes Mellitus

Perioperative adverse events were registered in 5 cases (29.4%) in the study group and in 8 (16.6%) in the control group (p>0.05). In the study group were recorded 2 cases of pulmonary and 3 of urinary bacterial infection treated with systemic antibiotic therapy. No case of thrombosis was registered. In the study group 4 patients (23.6%) required postoperative intensive care unit (ICU) for a mean of 5.3 days while in the control group 5 patients (10.4%) required postop ICU for a mean of 4.4 days (p>0.05).

## Discussion

The unprecedented, worldwide diffusion of the COVID-19 infection heavily affected the orthopedic trauma practice. Trauma surgeons all over the world are dealing with the uncertainty of treating COVID-19 positive patients, given the scarcity of literature data on perioperative risk. In this context, both clinical decisions and patient information lacks a solid scientific foundation. Therefore, we designed a study to evaluate the perioperative morbidity and mortality in COVID-19 trauma patients. In Italy as of November 23^rd^ 2020, 135,146 total cases have been identified since the first one, of which 47,654 death. Dying patients frequently present 3 or more comorbidities. The most common were hypertension and type-2 diabetes followed by heart diseases and chronic renal failure. The median age of dead patients was 48 years old [6].

Acute trauma in COVID-19 patients creates additional elements destabilizing the clinical picture and increasing the risk of adverse events. Hospitalization and reduction of mobility in cases with fractures requiring surgical procedure are indeed associated to respiratory complications. Furthermore, in the elderly, ASA grade ≥III is an additional risk factor for a complicated course [7], Catellani *et al*. reported the results of 16 elderly COVID-19 patients with a proximal femoral fracture. Seven patients died (3 before and 4 after surgery). The authors, in conclusion, did not recommend surgical treatment in COVID-19 patients with a significant reduction in oxygen saturation (<90%), fever (>38°C) and signs of systemic organ dysfunction [3]. Mi *et al*. analyzed the clinical course of 10 trauma COVID-19 patients with 6 of them over the age of 75; the authors found worse prognosis than in a traumatic patient. In the area with high incidence of infection they suggest conservative treatment of the fractures particularly in the elderly patients [8]. Egol KA *et al*. reported the results of 138 patients with mean age of 82.9 years with a hip fracture. Among these, 12.3% were COVID-19 positive and 10.1% suspected positive. They compared these two groups with negative patients. The authors underlined the high mortality in COVID-19 patients particularly if they needed ventilation [9]. It is well known that hip neck fractures in elderly persons are associated with excess mortality [10]. Our COVID-19 cohort of patients was characterized by a lower mean age (63.41 ± 18.77) and by higher heterogeneity of fractures. In authors opinion this could explain the better results in term of mortality. Moreover, also the patient death in our study needed for ventilation.

Muñoz Vives *et al*. reported results of 136 patients over 65 years old with proximal femoral fractures in a multicenter study. In the twenty-three positive cases, the mortality was 30.4% versus 10.3% of negative. Seven of the dead patients were COVID-19 positive and they had an ASA grade >III [11]. The different mortality in our study group (5.8%) could be related to anesthesiological complexity according to the ASA grade (average 2.35 ± 0.78). More to the point, the application of our COVID-19 trauma pathway allowed us to perform an early surgery in “the 72 golden hours”. The authors speculated that this dedicated management may be the strength underlying the good mortality results. The importance of early surgery in determining the prognosis of COVID-19 trauma patients was highlighted in other reports. This allows to reduce the risk of adverse events mainly due to bacterial super infections, respiratory diseases and deep-vein thrombosis [2,5,8]. The retrospective design is a limitation of our study. Further prospective studies with larger populations are needed to understand which could be the best management in COVID-19 trauma patients.

## Conclusion

In conclusion, our findings suggest that a dedicated COVID-19 trauma pathway with the aim of an early surgery could be key for a better result in terms of mortality and morbidity. Age and ASA grade could represent independent risk factors for perioperative complications.

### What is known about this topic

- SARS-CoV-2 virus from the Hubei region of China has spread all over the world becoming a pandemic disease;
- It is also not uncommon to find patients that access the emergency room for various diseases, including traumas and fractures, in which COVID-19 infection is moreover found;
- In literature there is a lack of clinical studies that evaluate prognosis and complications of COVID-19 positive patients with fracture treated with orthopedic surgery.

### What this study adds

- The application of our COVID-19 trauma pathway allowed us to perform an early surgery in “the 72 golden hours”;
- Our findings suggest that a dedicated COVID-19 trauma pathway with the aim of an early surgery could be key for a better result in terms of mortality and morbidity;
- Age and ASA grade could represent independent risk factors for perioperative complications.
